# High frequencies (HF) repetitive transcranial magnetic stimulation (rTMS) increase motor coordination performances in volleyball players

**DOI:** 10.1186/s12868-023-00796-2

**Published:** 2023-05-09

**Authors:** Fiorenzo Moscatelli, Giusi Antonia Toto, Anna Valenzano, Giuseppe Cibelli, Vincenzo Monda, Pierpaolo Limone, Nicola Mancini, Antonietta Messina, Gabriella Marsala, Giovanni Messina, Rita Polito

**Affiliations:** 1 Department of Clinical and Experimental Medicine, University of Foggia, Foggia, Italy; 2 Learning Science Hub, Department of Humanistic Studies, University of Foggia, Foggia, Italy; 3 Department of Movement Sciences and Wellbeing, University of Naples “Parthenope”, Naples, Italy; 4 Department of Human Sciences, Telematic University Pegaso, Naples, Italy; 5 Faculty of Physical Education and Sports, “Babes Bolyai” University, Cluj-Napoca, Romania; 6 Department of Experimental Medicine, Section of Human Physiology and Unit of Dietetics and Sports Medicine, Università degli Studi della Campania “Luigi Vanvitelli”, Naples, Italy; 7 Struttura Complessa di Farmacia, Azienda Ospedaliero-Universitaria di Foggia, Foggia, Italy

**Keywords:** Transcranial magnetic stimulation (TMS), Repetitive transcranial magnetic stimulation (rTMS), Dorsolateral prefrontal cortex (DLPFC), Interlimb motor coordination, Motor coordination, Cortical excitability, Athletes, Volleyball, Physical activity

## Abstract

**Introduction:**

It is widely demonstrated that high frequency (HF) repetitive transcranial magnetic stimulation (rTMS) has facilitative effects and is therefore capable to inducing changes in motor responses. One of the most investigated areas is the dorsolateral prefrontal cortex (DLPFC) as it plays a special executive attention role in actively preserving access to stimulus representations and objectives in environments with plenty of distraction such as those of team sports. Volleyball is a team sport in which the attention and coordination components are essential for achieving performance. Thus, the aim of this study was to investigate if HF rTMS at DLPFC in volleyball players can improve homolateral motor coordination and cortical excitability.

**Results:**

This study was a double-blinded (participant and evaluator) matched-pair experimental design. Twenty right-handed female volleyball players were recruited for the study and were randomly assigned either the active rTMS (n = 10) or the sham stimulation group (n = 10). The stimulation was performed in one session with 10 Hz, 80% of the resting motor threshold (RMT) of the right first dorsal interosseous muscle, 5 s of stimulation, and 15 s of rest, for a total of 1500 pulses. Before and after stimulation, the coordination and the cortical excitability were evaluated. The significant finding of this paper was that HF-rTMS of the DLPFC improved performance in terms of the homolateral interlimb coordination, with a significantly decreased in resting motor threshold and MEP latency of the ipsilateral motor cortex. It seem that HF-rTMS could increase coordination performances when the velocity of the execution is higher (120 bpm and 180 bpm).

**Conclusion:**

Moreover, in active rTMS group significant differences emerged after stimulation in RMT and in MEP latency, while no differences emerged after stimulation in MEP amplitude. In conclusion we believe that these results may be of great interest to the scientific community and may also have practical implications in the future.

## Introduction

The ability to pay attention is a crucial life skill that is intimately linked to memory, learning, perception, and executive function. The selection of a specific subset of the available stimuli and the simultaneous suppression of information that is currently irrelevant constitute the fundamental function of attention. The capacity for discrete response to visual or tactile inputs is known as focused attention. The ability to maintain a consistent behavioral reaction throughout ongoing and repetitive activities is known as sustained attention, while retain a behavioral or cognitive set in the face of competing or distracting stimuli is known as selective attention [1]. Alternating attention is a mental flexibility that enables people to change their point of focus and switch between tasks requiring various levels of cognition. Lastly, responding to numerous tasks or various task demands simultaneously is referred to as divided attention [2]. A fundamental component concerning selective attention is the ability to be able to shift attention from one place to another in relation to the different surrounding environmental situations. In particular, training induces persistent encoded behaviors within the adult nervous system [3, 4] to allow the precise execution of difficult motor tasks [5, 6]. Because it requires a high level of coordination for the precise execution of technical skills in static and dynamic conditions, athletes could represent a valuable model to investigate the effects of training on the corticospinal system excitability [7]. Expertise in a sport is the capacity to continuously display great athletic abilities. Although it is commonly acknowledged that elite athletes perform better than beginners, it is unclear if higher performance is the result of more skilled sensory-motor coordination. Athletes need to be able to recognize the visual fields that contain the most information, focus their attention in the right places, and efficiently and effectively extract meaning from these fields [7]. The most reliable indicator of anticipatory competence in team ball sports is the ability to recall and recognize an unfolding pattern of play. For instance, it is impossible to compare the anticipation of a volleyball serve, when the main objective is to send the ball over the net into the opposite court, with the anticipation of a setting action, where the aim is to place the ball in the ideal position for the assault [8]. As a result, it is feasible that players may in some circumstances solely rely on their ability to interpret information from an opponent's postural orientation, while in other circumstances they may be forced to make an anticipatory judgment based on perceived event probability. Even within the same sport, the sort of action being studied may have an impact on the capacity to draw out significant information from a sporting event [9].

An essential brain area, called the dorsolateral prefrontal cortex (DLPFC), plays a special executive attention role in actively preserving access to stimulus representations and objectives in environments with plenty of distraction [10] typical of team sports such as volleyball. In team sport, coordination has been considered ‘key to expert performance. In volleyball, the importance of coordination during the performance were positively associated with the teams’ success in major international tournaments [11]. Repetitive transcranial magnetic stimulation (rTMS) is a neuromodulation technique that makes use of electromagnetic coils placed on the scalp to create a magnetic field that, depending on the delivery settings, either stimulates or inhibits cortical activity. There is general agreement that rTMS below 1 Hz at the motor cortex lowers cortical excitability whereas rTMS over 5 Hz raises cerebral cortex excitability [12]. A systematic review found that high-frequency (HF) rTMS applied to the left DLPFC was most likely to result in selective cognitive improvement [13]. However, other studies show the following benefits: increased attentional control during the Stroop task, favorable effects of rTMS on attention in deficit hyperactivity disorder subjects, decreased reaction time [14], commission errors in a continuous performance test, improved working memory and coordination [15].

Our research hypothesis assumes that HF-rTMS of the DLPFC, having facilitating effects, can improve the coordination in volleyball players and increase cortical excitability. In fact, neurostimulation techniques to transiently modulate brain activity may offer crucial information to establish causal links between the left and right DLPFC and proactive control. Within this context, an important investigation [16] carried out an extensive literature review of the effects on different versions of the coordination and attentional task of HF-rTMS, a stimulation protocol that increases cortical excitability [17]. Based on this results, the authors proposed that the left DLPFC is active when there is foreknowledge of upcoming conflict, leading to coordination and attentional preparation. In contrast, the right DLPFC is proposed to participate in top-down attentional control when conflict is occurring at stimulus level.

Moreover, the TMS can be used to evaluate the excitability of the cerebral cortex using different stimulation protocol. These techniques include motor evoked potential (MEP) amplitude, resting motor threshold (RMT), and MEP latency [18]. After non-invasive brain stimulation, these instruments are known to be helpful for assessing the motor cortex's excitability and motor neurophysiology [17–24]. Thus the aim of this study was to investigate if HF-rTMS of the DLPFC in volleyball players can improve homolateral motor coordination and cortical excitability.

## Materials and methods

### Participants

This study was a double-blinded (participant and evaluator) matched-pair experimental design. Twenty right-handed [19] professional female volleyball players of Foggia and Cerignola (south of Italy) local team were recruited for the study and were randomly assigned either the active rTMS (n = 10) or the sham stimulation group (n = 10) (Table 1). The volleyball players were members of two local team, regularly competing at national levels and undergoing a training regimen of at least five 2-h sessions^.^week^−1^ for the previous 5 years. The local Institutional Ethics Committee approved the study (Azienda Ospedaliera-Universitaria “Ospedali Riuniti”, Foggia, Independent Ethics Committee; protocol number that was attributed by the ethics committee: 116/CE/2011, 14/11/2011). All subjects recruited for the investigation provided both written and oral information regarding the possible risks and discomforts and were ensured that they were free to withdraw from the study at any time. Furthermore, a medical examination ascertained the absence of psychoactive or vasoactive medication assumption, and risk factors or other contraindication according to the safety and recommendation for TMS use [20]. The subjects recruited for the study presented no contraindications and therefore no one was excluded from the procedure. During the 24 h preceding the start of the experimental procedures, the subjects recruited had to abstain from exercising and had to limit their caffeine intake. The subjects involved in the study were sent to report to the laboratory to be instructed on the experimental procedure. A detailed explanation was given regarding the performance of the interlimb motor coordination test and subsequently the evaluation of cortical excitability and related neurophysiological parameters (resting motor threshold = RMT; Motor evoked potential latency = MEP latency; motor evoked potential Amplitude = MEP amplitude). The rTMS procedure was purposely performed after the interlimb motor coordination test to avoid facilitation/inhibition effects resulting from this procedure. Immediately after the end of rTMS we again performed the evaluation of cortical excitability and the interlimb motor coordination test.

**Table 1:** The anthropometric parameters of the female volleyball players recruited for the study

Parameters	Active rTMS group N = 10	Sham group N = 10	p value
Age (years)	22.0 ± 2.9	22.0 ± 3.8	p > 0.05
Height (cm)	173.0 ± 5.0	171.7 ± 8.2	p > 0.05
Body mass (Kg)	66.1 ± 9.5	61.8 ± 3.6	p > 0.05

No significant differences emerged between “Active rTMS group" and "sham group"

### Study design

The subjects recruited for the study were randomly divided into four groups and invited to the physiology laboratory of the University of Foggia on four consecutive days. Upon entering the laboratory, each subject was explained the entire experimental procedure which began after the signing of the informed consents. Before the beginning of the session the subjects were explained how to carry out the motor coordination test. To ensure that all study subjects understood how to perform the coordination test, a non-study subject showed them how to perform the test. The subjects did not perform coordination tests to avoid a training effect that could have distorted the results, and it was preferred to verify directly the skillful ability of the players using a new task. Subsequently the tests were performed with this temporal sequence: detection of anthropometric parameters, motor coordination tests, positioning on the chair and identification of the RMT and recording of twenty stimuli for MEP analysis, HF-rTMS and, immediately after the end of the stimulation, the subjects performed again the motor coordination test and the recording of twenty stimuli for analysis of the MEP.

### TMS and electromyographic recording

To minimize any possible circadian influence, the recording session were performed between 9:30 AM and 12:30 AM. With the subject sitting on an armchair in a quiet room, motor cortex excitability was tested using a Magstim^®^ Rapid device (Magstim Co., Ltd., Whitland, South West Wales, United Kingdom) with an 80-mm figure-of-eight coil placed over the left motor cortex. A mechanical arm maintained the handle of the coil tangential to the scalp with the handle pointing backward at 45° away from the midline while delivering stimulus [21, 22]. The head of each subjects was secured to the sit and throughout the procedure they remain completely relaxed. The location of the stimulation was identified on each subject’s scalp using the SofTaxic navigator system (E.M.S. Italy, http://www.emsmedical.net). The RMT was determined as the minimum stimulator intensity that evoked a peak-to-peak motor evoked potential of > 50 µV in at least 5 out of 10 consecutive trials [23, 24]. After the identification of the rMT, 20 stimuli were collected for each subject. Following TMS, MEP latencies (i.e., the velocity at which the neural signal is propagated from the motor cortex to the muscle) and amplitudes (i.e., the magnitude of corticospinal excitability) at 120% of RMT, were measured by means of surface electromyography (EMG) recordings (Biopac MP150, BIOPAC Systems, Inc., CA, USA) in the first dorsal interosseus (FDI) muscle of the right hand. Using a classical belly-tendon montage, surface pre-gelled disposable electrodes (Biopac system, snc; 1 cm, diameter) were placed in correspondence of the FDI muscle (active electrode) and over the associated joint or tendon (reference electrode), whereas the ground electrode was placed on the dorsal part of the forearm. The electrodes incorporate liquid electrolyte gel and moderately-high chloride salt concentration. The magnetic stimulator was connected to the PC, and interfaces with the EMG recording software. The stimulator sends a square wave signal (Trigger) each time it is activated. So on the EMG trace first was shown the trigger and immediately after the muscle response. The latency time was considered the time between the trigger itself (onset of the square wave) and the start of muscle response. Raw EMG signals were processed and analyzed (Acknowledge software, version 4.1, BIOPAC Systems, Inc., CA, USA) with a high pass filter (cutoff frequency: 10 Hz). For RMT condition, twenty responses were averaged. We tested the protocol on left cortex (M1) for all subjects.

### rTMS protocol

**Fig. 1:**
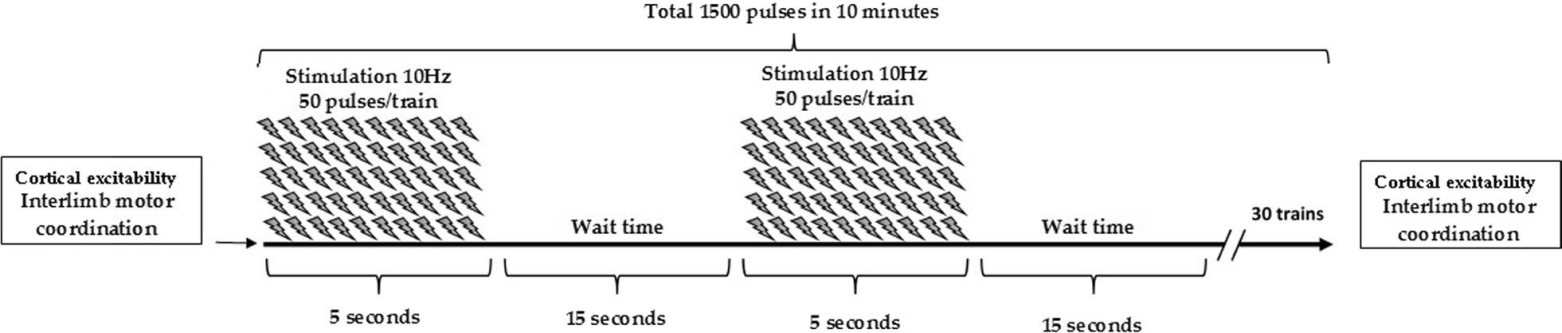
The study protocol. After cortical excitability investigation and interlimb motor coordination test, the volleyball players assigned to the stimulation group performed rTMS at 10 Hz, 80% of the RMT of the right first dorsal interosseous muscle, 5 s of stimulation, and 25 s of rest, for a total of 1500 pulses

The rTMS was delivered to the left DLPFC, which is defined as channel F3 according to the international 10–20 system. The coil was held with the handle posterior and oriented sagittally. The subjects were seated in a comfortable chair. The stimulation was performed in one session with 10 Hz, 80% [23] of the RMT of the right first dorsal interosseous muscle, 5 s of stimulation, and 15 s of rest, for a total of 1500 pulses (Fig. 1). Sham stimulation was performed in the same manner except that the coil was held at an angle of 90◦, and only one edge of it rested on the scalp. During rTMS, all participants wore earplugs, and safety guidelines were followed [20].

### Interlimb coordination performance

**Fig. 2:**
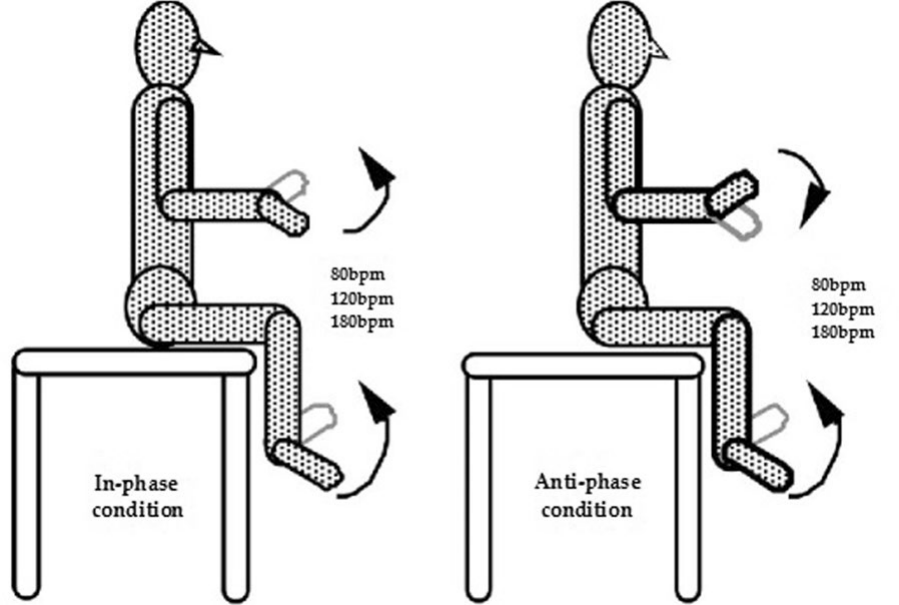
In-phase condition mode (associations of wrist extension with ankle dorsal flexion and wrist flexion with ankle plantar flexion) and anti-phase condition mode (association of wrist flexion with ankle dorsal flexion and wrist extension with ankle plantar flexion)

Homolateral hand and foot coordination was evaluated by means of a validated field test [24], which proved to discriminate the effects of training in situational [24–27] and closed skill sports [25]. The participants were seated shoeless on a table with elbows and knees flexed at 90 degrees. Observing the spatial and temporal constraints of the movement patterns, they had to perform cyclic flexion and extension movements around the wrist and ankle joints with a 1:1 ratio. Two homolateral coordination modes were tested: in-phase (IP) (i.e., association of hand extension with foot dorsal flexion and hand flexion with foot plantar flexion) and anti-phase (AP) (i.e., association of hand flexion with foot dorsal flexion and hand extension with foot plantar flexion). Each test condition was performed at 3 different frequencies (80, 120, and 180 bpm, respectively) dictated by a metronome for a total duration of 60 s. During the 2-min rest between test trials, the subjects were allowed to stand. Following 15 s of the required metronome pace, a ‘‘ready-go’’ command indicated the start of a trial. Using a stopwatch, an observer measured the time (s) of correct execution from the beginning of the movement until the subject failed to meet either the spatial and/or the temporal task requirements. To avoid disagreement among observers, a single competent observer scored the inter-limb performances (Fig. 2). Each subject therefore followed the coordination test in both in-phase and anti-phase modalities; moreover, for one of the two modalities he performed the test at all frequencies, i.e. 80 bpm, 120 bpm, 180 bpm. Furthermore, the different modalities were subjected to the subjects in a random way to avoid a training effect.

### Statistical analysis

Statistical analyses were performed by the GraphPad 6 Software, Inc., for Windows, version 6.01. The data are presented as mean (M) ± standard deviation (SD), and statistical significance was set at *p* < 0.05. The Shapiro–Wilk test was used to check the normal distribution of variables. The differences in antropometric parametres between the active and sham rTMS groups were analyzed with an independent t-test. A 2 (Groups: Athletes, Controls) × 2 (Coordination Mode: IP, AP) × 3 (Execution Frequency: 80, 120, 180 bpm) ANOVA for repeated measures was applied to the time (s) of correct execution of the inter-limb coordination test. If the overall F test was significant, Tukey’s post-hoc comparisons were used. To test the significant difference changes in MEPs parameter (active rTMS vs. sham) an independent t-test was used.

## Results

### Interlimb motor coordination test

No discomfort or adverse effect were reported during and after TMS procedure. The interlimb motor coordination test was performed before and after active and sham stimulation (Table 2).

**Table 2:** Effects of HF-rTMS on the interlimb motor coordination test

Parameters	Active group			Sham group		
Bpm/condition	Pre	Post	Δ	Pre	Post	Δ
80 IP	54 ± 11	54.4 ± 8.38	0.4 ± 6.25	49.1 ± 16.954	48.8 ± 13.677	− 0.3 ± 9.31
120 IP	15.2 ± 5.3	44.3 ± 9.82	29.1 ± 9.02	29.4 ± 9.3238	25.1 ± 15.118	− 4.3 ± 16.75
180 IP	12.6 ± 5.6	21 ± 4.85	8.4 ± 4.97	14.6 ± 6.8508	14.1 ± 4.4083	− 0.5 ± 7.16
80 AP	53.3 ± 15	57.3 ± 3.62	4 ± 11.47	46 ± 15.684	45.4 ± 17.36	− 0.6 ± 10.42
120 AP	10.9 ± 4.3	23.7 ± 6.25	12.8 ± 8.05	14.3 ± 9.4522	14.9 ± 5.6263	0.6 ± 5.27
180 AP	4.9 ± 2.4	9.4 ± 3.20	4.5 ± 2.46	5.1 ± 3.9001	5.1 ± 2.1833	0 ± 2.21

*IP*  in phase, *AP* anti-phase

**Fig. 3:**
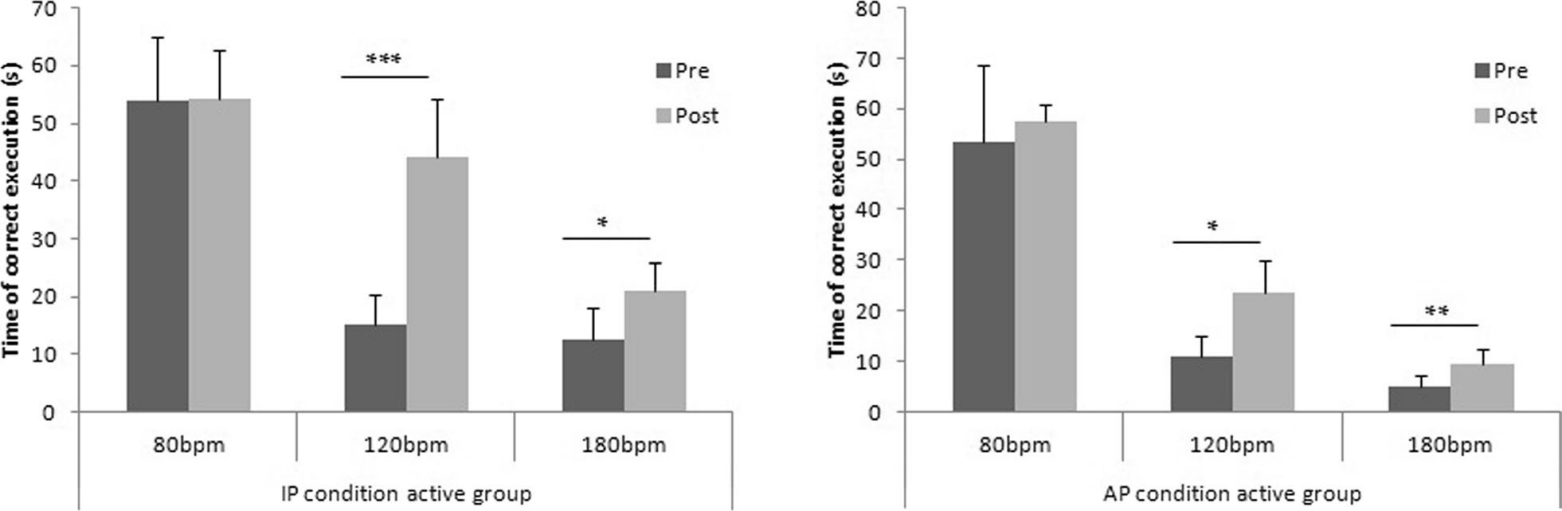
The time of correct execution of interlimb motor coordination test (in-phase/anti-phase) in active rTMS group. *p < 0.05; **p < 0.01; ***p < 0.001

The ANOVA multiple comparison show significant differences in active group (F(2.560, 23.04) = 109.3; p < 0.001). Tukey’s multiple comparisons test show significant differences in both condition (IP/AP) at 120 bpm and 180 bpm. In particular, in IP condition, volleyball players increase their coordination performance after HF stimulation at 120 bpm (p < 0.001) and at 180 bpm (p < 0.05). Moreover significant differences emerged also in AP condition at 120 bpm (p < 0.05) and at 180 bpm (0.01), while in both condition (IP/AP) no significant differences emerged at 80 bpm (p > 0.05) (Fig. 3).

**Fig. 4:**
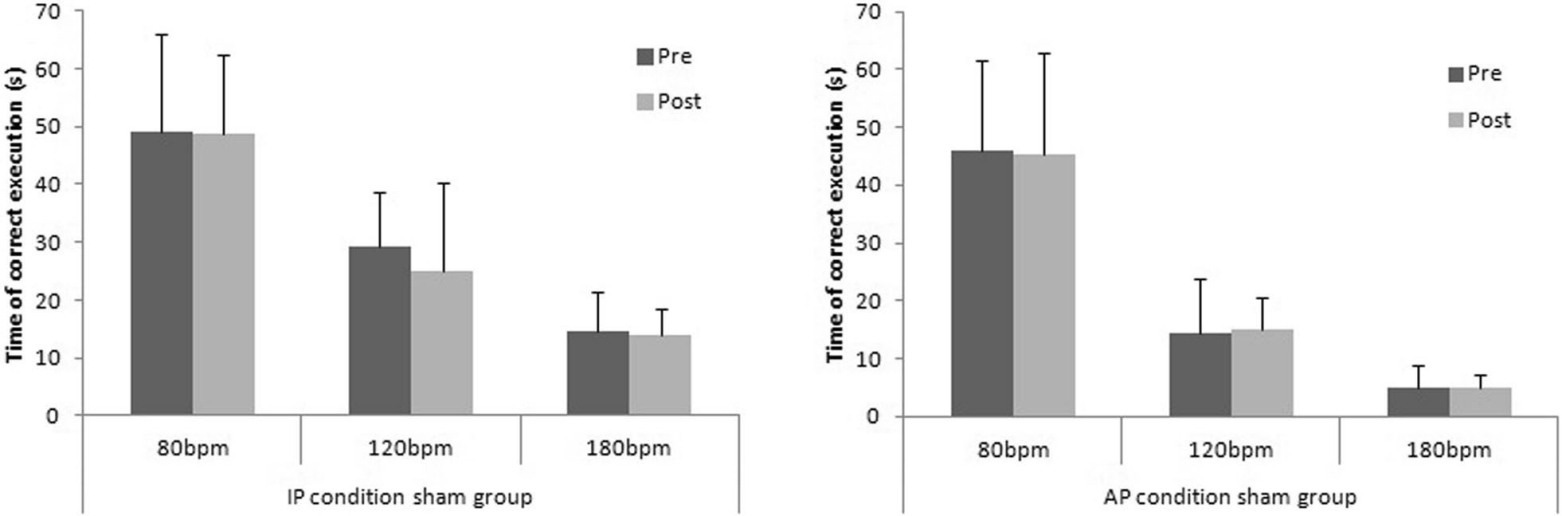
The time of correct execution of interlimb motor coordination test (in-phase/anti-phase) in sham rTMS group

The ANOVA multiple comparison show no significant differences in sham group (F(2.731, 24.58) = 109.3; p < 0.05). Tukey’s multiple comparisons test show no significant differences in both condition (IP/AP) at 80 bpm, at 120 bpm and 180 bpm (Fig. 4).

### MEPs

**Fig. 5:**
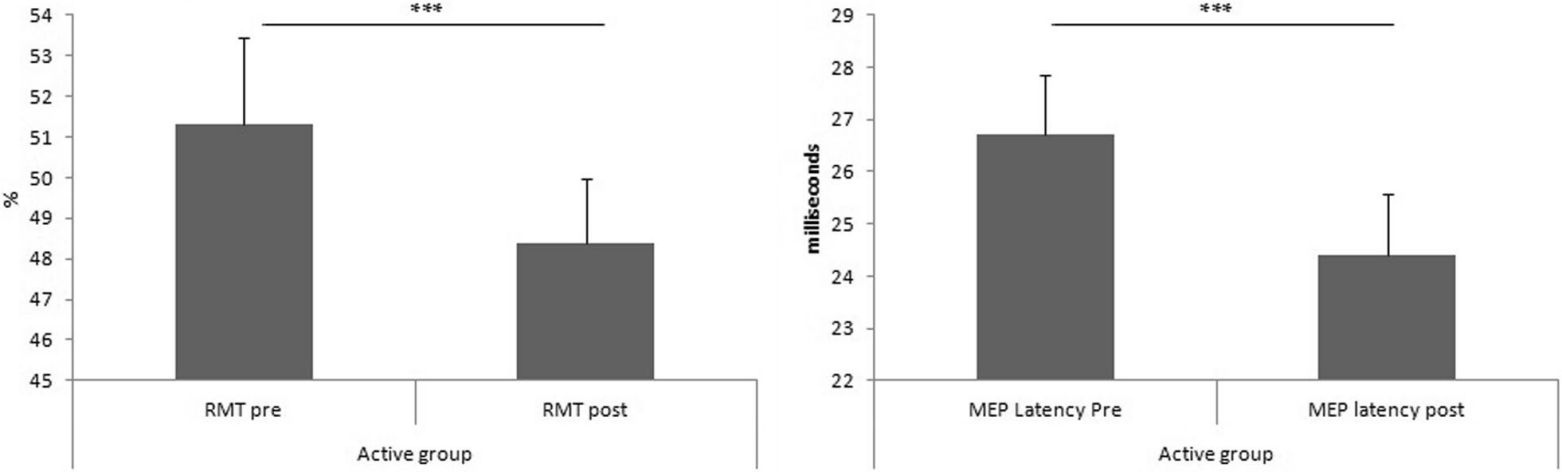
The differences between RMT and MEP latency before and after HF rTMS. *RMT* resting motor threshold; *MEP* motor evoked potential; *HF* high frequencies; *rTMS* repetitive transcranial magnetic stimulation. *** p < 0.001

In active rTMS group the RMT changed from a starting mean value 51.3% (± 2.16) before the stimulation to a mean value of 48.4% (± 1.57) (p < 0.001). Moreover, in active rTMS group the MEP latency changed from a starting mean value 26.7 ms (± 1.15) before the stimulation to a mean value of 24.4 ms (± 1.17) (p < 0.001) (Fig. 5). Finally, always in active rTMS group, the MEP amplitude changed from a starting mean value 0.72 mV (± 0.09) before the stimulation to a mean value of 24.4 ms 0.73 mV (± 0.09) (p > 0.05). The *t*-test show no significant differences in RMT, MEP latency and MEP amplitude before and after sham stimulation.

## Discussion

The significant finding of this paper was that HF-rTMS of the DLPFC seems to improve performance in terms of the homolateral interlimb coordination, with a significantly decreased RMT and MEP latency of the ipsilateral motor cortex. After stimulation, in active group increase the time of correct execution of the interlimb motor coordination test in both condition (in-phase/anti-phase). It seem that HF-rTMS could increase coordination performances when the velocity of the execution is higher (120 bpm and 180 bpm). Moreover, in active rTMS group significant differences emerged after stimulation in RMT and in MEP latency, while no differences emerged after stimulation in MEP amplitude. Our results are in line with previously published paper. In fact, different authors, reported changes in the neurocognitive profile after HF-rTMS of the left DLPFC [2]. In an important systematic review were reported that, rTMS of the DLPFC, significantly improved all measures of working memory, performance and coordination [15]. In our work, HF-rTMS increase coordination performances and cortical excitability. There are several possible reasons for this. To execute movement, the target muscle activation underlying movement is controlled by the central nervous system [28]. It is well understood that the primary motor area M1, encodes execution aspects such as movement trajectory and force generation. Furthermore, it has been established that the DLPFC is responsible for visuospatial processing through its connection to the dorsal stream of visual pathway [29]. These processes rely on high-level awareness of the world being perceived, requiring the brain to integrate visual input information and translate it into an actual output motor plan. Both the study of visual perception and the study of motor perception and coordination are multidisciplinary fields that integrate knowledge and data from the three disciplines of neuroscience, psychology, and computation. The information splits into two processing streams in the primary visual cortex at an early stage of cortical visual processing. The inferior parietal lobule's posterior half is reached by the dorsal stream, which then travels through the occipitoparietal cortex to the DLPFC [30]. In the medial-temporal lobe, there are two distinct groups of cells: visuomotor cells, which are only associated with the kinematics of actual motions when visual information is present, and motor-like neurons, which are involved in hand movements even when visual input is absent. This study focuses on an early stage in the process of sensorimotor transformations: the transformation between visual input and hand kinematics (output), which comes before known transformations between extrinsic and intrinsic coordinate frames of the acting limb. The authors found a population of visuomotor speed- and direction-related neurons in the parahippocampal gyrus [31].

Regarding the physiology of HF-rTMS over DLPFC, prior research indicated that a single session of HF-rTMS caused the ipsilateral head of the caudate nucleus in the striatum to release dopamine. The dopamine neurons in the striatum were affected by the DLPFC stimulation because it improved the efficiency of the glutamate neurotransmitter and glutamate receptors. Additionally, some glutamate synaptically connected with medium spiny neurons that were almost in the ventral tegmental region (VTA) via their dendritic spines. This process aided in controlling the release of dopamine in the VTA because of the stimulation of dopamine neurons in the region. Therefore, HF-rTMS of the DLPFC may result in an enhancement in visuospatial processing and consequently improvement in motor performance [32]. In another important investigation the authors observed improvement in temporal coordination in the barrier condition after HF stimulation of DLPFC. These findings support the role of DLPFC in visuospatial processing and coordination of movement [29].

Additionally, there is mounting evidence that the DLPFC controls simple motor activities top-down in addition to its role in higher order executive processes, particularly in the temporal arrangement of goal-oriented behaviors. During directed finger reaction movements, authors demonstrated a selective regulation of M1 excitability (time- and muscle-specific) during movement preparation with origin in the DLPFC. Brain imaging reveals functional connection between the left DLPFC and sensorimotor cortices in the case of simple repetitive movements [32]. Surprisingly, efficient analyses of connectivity that reveal the direction of interactions between the left DLPFC, premotor, and sensorimotor cortices support DLPFC top-down control over those motor structures.

A recent study has investigated the effect of HF-rTMS over the left and right DLPFC on the coordination performance. After HF-rTMS over the left DLPFC, the authors observed an increase in proactive cognitive control when compared to the performance after the sham stimulation and after right DLPFC HF-rTMS. The results of this study provide experimental evidence on the role of the left DLPFC in proactive cognitive control processes. Moreover, this findings suggest that the right DLPFC may be implicated in reactive control [16].

In the present study, the RMT and the MEP latency decreased after HF-rTMS of the DLPFC, which indicates increased motor cortex excitability. The important finding from our study is that non-invasive brain stimulation in the left DLPFC did not prevent excitability from rising in the M1 motor cortex. These results lend credence to the idea that prefrontal rTMS may influence corticomotor excitability indirectly and enhance cognitive performance by causing even more remote changes in the cortical and subcortical systems. The automaticity of motor function and its cognitive function are both governed by connections between the basal ganglia and frontal lobes, and motor cognition includes both cortical and subcortical components [33]. According to previous published study, the M1 cortex and frontal lobe have a physically hierarchical relationship, and the frontal lobe controls cognitive function. Before and during the execution of sequential motor activity, other investigators observed single-cell activity in the M1 and cognitive region of monkeys' frontal lobes [34]. They showed that the frontal lobe and M1 cortex had intricate physiological relationships and interactions [35]. According to Friston (2002), the context-dependent interactions between various brain regions based on exact anatomical and functional connectivity underlie the sensory, motor, or cognitive processes [36]. This lends credence to the idea that the frontal lobe, which controls cognitive function, is physically and functionally linked to the motor cortex.

## Conclusions

Our study shows that a single session of HF-rTMS of the DLPFC in volleyball players seems to improve coordination and cortical excitability. These results could provide useful tools to modulate sports training. In fact, these results, if confirmed, could lead trainers to offer their athletes rTMS sessions suitably blended with training. However, despite the interesting results, the study has some limitations such as a small sample, that should be increasing and investigated in the future to clarify all aspects. Furthermore, the effects of rTMS at different frequencies should also be evaluated (in our study we performed rTMS only at 10 Hz) to establish the best protocol to obtain an improvement in performance. Furthermore, a study including male athletes and non-athletes should also be conducted. In conclusion, despite the limitations described above, we believe that these results could be of great interest to the scientific community and they could have practical implications in the future.

## Data Availability

All data generated or analysed during this study are included in this published article.
